# RETRACTED: Iftikhar et al. Endogenous Retroviruses as Regulators of Innate Immune Signaling and Inflammation. *Viruses* 2026, *18*, 289

**DOI:** 10.3390/v18080835

**Published:** 2026-07-29

**Authors:** Muhammad Iftikhar, Xinyan Wang, Qiangzhou Wang, Jiaxing Wang, Lihong Gu, Shihao Chen

**Affiliations:** 1 College of Animal Science and Technology, Yangzhou University, 48 East Wenhui Road, Yangzhou 225009, China; iftikhark34@gmail.com (M.I.); 2869623152@qq.com (Q.W.); nil2008@yeah.net (L.G.); 2 Institute of Animal Science & Veterinary, Hainan Academy of Agricultural Science, Haikou 571700, China; wxy_20060704@qq.com; 3 College of Veterinary Medicine, Yangzhou University, Yangzhou 225009, China; 1508466288@qq.com

*Viruses* retracts the review article titled “Endogenous Retroviruses as Regulators of Innate Immune Signaling and Inflammation” [1], cited above. 

Following publication, concerns were brought to the attention of the publisher regarding an overlap between this publication [1] and a previously published review article [2], produced by a different author group.

In accordance with the journal’s standard procedures, an investigation was conducted by the Editorial Office and Editorial Board that confirmed a significant level of overlap between these two publications [1,2]. Given the extent of the overlap, the Editorial Board has decided to retract this article as per MDPI’s retraction policy.

This retraction was approved by the Editor-in-Chief of *Viruses*.

The authors have been informed of this retraction and have indicated their agreement with this decision.
